# Neoadjuvant Treatment Versus Upfront Surgery in Gastric Cancer Patients: Early Postoperative and Pathological Results: A Retrospective Study in a Tertiary Center

**DOI:** 10.3390/jcm15062342

**Published:** 2026-03-19

**Authors:** Bogdan Filip, Ana Grigoraș, Dragos-Viorel Scripcariu, Mihaela-Mădalina Gavrilescu, Dragoș Predescu, Viorel Scripcariu

**Affiliations:** 1 Deparyment of Surgical Sciences, Faculty of Medicine, Grigore T. Popa University of Medicine and Pharmacy, 700115 Iasi, Romania; bogdan.filip@umfiasi.ro (B.F.); gavrilescu.mihaela@umfiasi.ro (M.-M.G.); viorel.scripcariu@umfiasi.ro (V.S.); 2 I-st Oncological Surgical Unit, Regional Institute of Oncology, 700483 Iasi, Romania; 3 Deparyment of Surgical Sciences, Faculty of Medicine, Carol Davila University of Medicine and Pharmacy, 050474 Bucharest, Romania; drpredescu@yahoo.com; 4 General and Esophageal Surgery Department, Sf. Maria Clinical Hospital, 011172 Bucharest, Romania

**Keywords:** gastric cancer, radical gastrectomy, upfront surgery, neoadjuvant treatment, surgical safety

## Abstract

**Background/Objectives:** Gastric cancer remains one of the most frequent abdominal malignancies, being the 5th in incidence, and it is often diagnosed at advanced stages. Perioperative chemotherapy has been introduced to improve oncological outcomes, although concerns persist regarding increased postoperative complications after radical gastrectomy following neoadjuvant treatment (NAT). **Methods:** We performed a retrospective study on a continuous series of gastric cancer patients who underwent radical gastrectomy between January 2016 and December 2025. Patients were divided into two groups: those receiving NAT and those undergoing upfront surgery (US). Demographic data, clinical characteristics, operative variables, postoperative complications, 30-day mortality, and pathological findings were compared. **Results:** There were 383 patients included in the study; NAT was performed in 105 (27.4%) cases and US in 278 (72.6%) cases, with a mean age of 64.99 (63.09–66.88) vs. 67.83 (66.44–68.91) years. Baseline characteristics (Charlson score 3.8 vs. 4.26, *p* = 0.131), hemoglobin, and albumin levels, were similar between groups. Tumors in the NAT group were more frequently located in the upper stomach 19 (18.1%) vs. 33 (11.9%) or at the gastro-esophageal junction (only Siewert III tumors were included) 17 (16.2%) vs. 23 (8.3%) *p* = 0.04. Tumor stage was most frequently stage IIIB for radical surgery 111 (28.9%) and for the NAT group 20 (19.1%) and surgery first group 91 (32.7%). The overall grade III and above complication rates were 26 (6.8%) esojejunal fistula, 19 (4.9%) duodenal stump fistula, seven (1.8%) hemorrhagic complications, 31 (8.1%) cases of sepsis, and 33 (8.6%) medical complications. Anastomotic fistulas were more frequent in the surgery-first group, with 23 cases (8.3%), compared with the neoadjuvant group, with 3 patients (2.9%)—the result were not statistically significant. The number of resected lymph nodes was similar (35.4 vs. 35.2; *p* = 0.96), while NAT group had significantly fewer positive lymph nodes (5.7 vs. 8.0; *p* < 0.001). Complete pathological response was achieved in 10 (9.5%) of NAT patients. **Conclusions:** Neoadjuvant treatment does not appear to increase the complications rate following radical gastrectomy. This study supports the surgical safety of the perioperative adjuvant treatment for advanced gastric cancer patients. Further studies are necessary to assess long-term outcomes.

## 1. Introduction

Gastric cancer remains one of the most frequent tumors; it was estimated that in 2022 there were almost 969,000 new cases, making it the 5th in incidence, accounting for about 4.7% of all new cancers [1]. There are regional differences in incidence, influenced by factors like Helicobacter pylori infection, diet particularities, genetic factors and the screening programs. The highest incidence is encountered in Eastern Asia (with an incidence of 32.1 per 100,000 among males and 13.2 among females) countries and the lowest in North America (5.6 per 100,000); Eastern Europe has higher rates, above the global average [2]. Currently, the best treatment for gastric cancer remains surgical resections, in the absence of metastatic disease. In case of metastatic disease, palliative chemotherapy remains the only treatment option. There is no single best treatment for gastric cancer; the actual trend in cancer care is personalized treatment, which is usually discussed by a multidisciplinary tumor board. The choice of treatment depends on stage, tumor location (proximal, distal or gastro-esophageal junction), tumor biology (HER-2, PD-L1, MSI/MMR) and the patient’s general condition [3].

For resectable disease (stage I–III), the standard approach in Western Countries represents the choice between perioperative chemotherapy and radical gastrectomy followed by adjuvant chemotherapy. According to the current recommendations for systemic treatment in gastric cancer, patients with locoregional disease (cM0, any N), who are medically fit and have potentially resectable tumors classified as cT2 or higher, should receive perioperative systemic therapy (category 1). In cases of MSI-H/dMMR tumors, neoadjuvant or perioperative immune checkpoint inhibitors may also be considered. Therefore, the neoadjuvant treatment referred to in our study is generally part of a broader, multimodal therapeutic approach [4]. The rational for neoadjuvant chemotherapy is to reduce the size of the tumor, to increase the probability of complete resection, to reduce the risk of recurrence and nevertheless to see the response to treatment (to select the tumors resistant to therapy). The benefits of the neoadjuvant treatment are: higher R0 resection rate, better survival of the patients and better systemic control of the disease. There are some downsides of this approach such as: systemic toxicity of chemotherapy, and a percentage of patients will not be able to sustain further surgical intervention. There were several major clinical studies with a high level of evidence that shaped the introduction of neoadjuvant treatment in gastric cancer: the MAGIC trial (1996–2002) was the first chemotherapy trial that compared surgery alone versus perioperative epirubicin, cisplatin and fluorouracil in patients with resectable gastric cancer and gastroesophageal junction adenocarcinoma [5], and it showed an improved 5-year survival with chemotherapy from 23% to 36%. A French trial (FFCD9703) compared perioperative cisplatin and fluorouracil versus surgery alone in locally advanced gastric cancer and confirmed the improved 5-year overall survival from 24 to 38%, with a higher R0 resection rate and better disease-free survival [6]. The FLOT 4 trial compared perioperative FLOT (fluorouracil, leucovorin, oxaliplatin and docetaxel) versus ECF/ECX (epirubicin-bases regimens) in patients with resectable gastric or gastroesophageal junction adenocarcinoma, and it showed an increased pathological complete response rate with a longer median overall survival (50 months) and improved median disease-free survival (30 vs. 18 months) with FLOT [7]. Based on the results of this study, FLOT became the preferred perioperative regimen in fit patients due to superior survival and response rates. All those benefits in terms of improved overall survival and disease-free survival were confirmed by multiple meta-analyses [8,9].

Radical gastric cancer surgery, especially total gastrectomy, remains a highly demanding surgical intervention with potential severe morbidity and mortality. Overall complication rate varies around 20–30% with variations based on definition, classifications and reporting standards [9,10], with a non-neglectable death rate of 2–3% [11]. Most common complications include anastomotic leak, pulmonary infections, pancreatic fistula and hemorrhage and abdominal abscess. There were several studies that showed that neoadjuvant treatment does not significantly increase overall postoperative complications rate. A meta-analysis of randomized trials comparing neoadjuvant chemotherapy plus surgery to upfront surgery found no significant increase in overall postoperative complications, pulmonary complications, anastomotic leakage, surgical site infections, or perioperative mortality with neoadjuvant treatment [12]. Population-based data showed similar 90-day complication rates in patients who received neoadjuvant therapy versus those who went straight to surgery (40.7% vs. 42.4%). Major complications (Clavien-Dindo ≥ III) were also similar (17.9% vs. 16.3%) [13]. There are also some studies that show that the FLOT regimen is associated with fewer major surgical complications and reinterventions [14].

The aim of our study is to evaluate the results of the neoadjuvant therapy regimens in a tertiary referral center for gastrointestinal malignancies and to assess the early postoperative outcomes following the perioperative treatment for gastric cancer.

## 2. Materials and Methods

We performed a retrospective study on a prospective collected database that included all patients for whom surgery was performed for gastric cancer in the 1st Surgical Unit of Regional Institute of Oncology, Iasi. We selected all consecutive gastric cancer patients from January 2016 until December 2025 who underwent radical surgery. The inclusion criteria were: adult patients aged 18 years or older, with histopathological confirmed gastric adenocarcinoma, in which complete clinical, staging, operative data and postoperative course was obtained. We excluded other gastric tumor pathological subtypes, such as lymphoma, neuroendocrine tumors, sarcoma or gastrointestinal stromal tumor. We also excluded patients with hepatic or distant metastasis. Although most of these patients underwent palliative procedures—and in selected cases, palliative gastrectomy was performed—they were not included in the analysis.

All patients underwent preoperative staging with computed tomography of the chest, abdomen, and pelvis. Magnetic resonance imaging was not routinely performed and was reserved for selected cases for research purposes. Patients with imaging findings suspicious for distant lymph node involvement (e.g., enlarged mediastinal lymph nodes) or other distant metastasis underwent positron emission tomography (PET). This protocol was also applied to patients considered for neoadjuvant therapy to exclude distant disease and to optimize selection of those who might benefit from radical resection.

The decision to administer neoadjuvant therapy was made during a multidisciplinary tumor board that included the operating surgeon, a dedicated gastrointestinal oncology specialist, a radiologist, and a radiation oncologist. The decision-making algorithm was based on the tumor’s clinical characteristics (such as location, symptoms, or complications), the clinical stage determined by preoperative imaging, and the patient’s comorbidities and preferences. Neoadjuvant treatment was generally favored for patients with advanced tumors, clinical evidence of regional lymph node metastasis—particularly in nodal stations beyond the planned resection field—or suspected local invasion or presumed peritoneal carcinomatosis. Neoadjuvant chemotherapy protocol included: FLOT regimen (Docetaxel, Oxaliplatin, leucovorin and 5-fluorouracil), ECF regimen (epirubicin, cisplatin and 5-fluorouracil) or DCX regimen (docetaxel, cisplatin and capecitabine). The number of cycles varied between 3 and 6 based on patients’ clinical status, complications and imaging evaluations. The neoadjuvant protocol did not include radiotherapy; only in selected cases with hemorrhagic tumors without response to conservatory treatment was a short course of radiotherapy used for hemostatic purposes, and subsequently the patients were submitted to chemotherapy protocol.

After the completion of the chemotherapy protocol, the patient was restaged using a tomography of the thorax, abdomen and pelvis, and also in selected cases a PET-scan was performed to exclude distant metastasis. Based on the clinical and imaging results, a decision to submit the patient to radical surgery was performed.

Prior to surgery all the patients had a complete evaluation of the comorbidities with focus on the cardiovascular status, especially the patients that underwent neoadjuvant chemotherapy, due to the cardiotoxicity effect of the chemotherapy regimens. Patients with severe weight loss and malnutrition were admitted prior to surgery for a structured nutritional support protocol and anemic patients with Hb levels below 7.5 d/dL required blood transfusion prior to surgery or intravenous administration of iron derivates. Surgery was performed by 4 surgeons with experience and expertise in oncologic surgery. Prior to the induction of anesthesia, a peridural analgesic catheter was placed based on the anesthesiologist’s choice. All patients underwent radical gastrectomy with D2 lymphadenectomy. The choice between total and subtotal gastrectomy was based primarily on the location on the tumor and the ability to obtain negative resection margins. In cases of total gastrectomy, splenectomy was performed based on tumor location (for example tumors of the great curvature in the vertical portion) or on the surgeon’s experience. For tumors of the gastroesophageal junction, the surgical approach consisted of either a median laparotomy and a separated lower left thoracotomy or an oblique thoracoabdominal incision. Reconstruction following radical gastrectomies consisted of Roux-en-Y esophago- or gastrojejunostomy. In patients with total gastrectomy, a feeding jejunostomy catheter was placed and the patient remained a jeun for 7 days or longer based on clinical course, and after the contrast studies of the integrity of the anastomoses, a liquid diet was initiated followed by solid foods. Complications following surgery were analyzed based on the Dindo-Clavien classification [15], in minor and major complications, and we also assessed 30-day mortality. We performed a univariate analysis of variables involved in the appearance of a postoperative complication. Statistically significant variables were introduced in a multivariate logistic analysis.

We divided the patients into two subgroups: the first one included the cases in which neoadjuvant treatment was performed, and the other included the patients in which surgery was the first treatment sequence. We assessed patient’s clinical and tumoral characteristics, the comorbidities (coded according to the Charlson comorbidity scale) [16], operative findings and procedure, postoperative course in terms of morbidity and mortality and the final pathological result of the surgical specimen. The results were analyzed using MedCalc version 23.4.8 (MedCalc Software, Ostend, Belgium). We used descriptive statistics to summarize the group characteristics; to compare continuous variables, the Mann–Whitney U test was used, and for the categorial variables, the chi-square and Fiser exact test were used. All results were interpreted with a 95% confidence interval, and a *p* value <0.05 was considered statistically significant.

The study was conducted with approval from the University of Medicine and Pharmacy in Iasi, with compliance with the ethical standards of the institutional research committee, and all the patients provided a written informed consent at the beginning of the treatment.

## 3. Results

**Figure 1:**
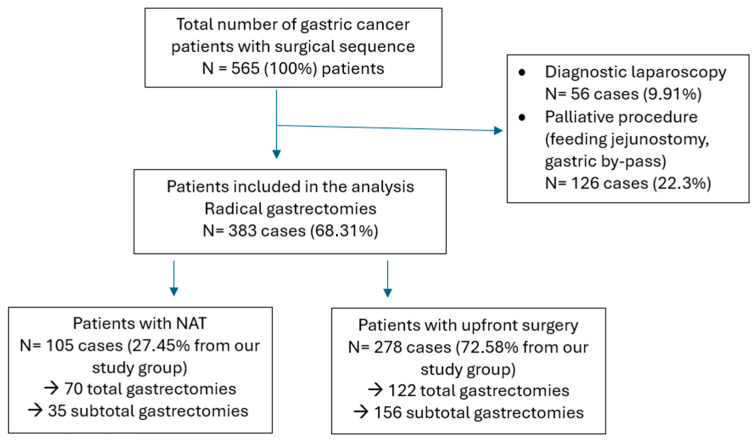
Study selection—Flowchart.

During the study period there were 565 patients diagnosed with gastric cancer admitted in the 1st Surgical Unit of the Regional Institute of Oncology, Iasi. In 383 (68.31%) cases radical surgery was performed; for the rest of the patients, they received either a diagnostic laparoscopy to confirm peritoneal carcinomatosis, which occurred in 56 (9.91%) cases, or a palliative procedure, which was in 126 (22.3%) cases (feeding jejunostomy, gastric by-pass for gastric outlet obstruction). As shown in Figure 1, there were 105 (27.45%) patients in which neoadjuvant treatment was performed following radical surgery, and 278 (72.58%) patients in which surgery was the first treatment option.

The patients’ characteristics are summarized in Table 1. There was no difference in terms of mean age—64.99 (63.09–66.88) years for the neoadjuvant treatment group versus 67.83 (66.44–68.91) for the surgery-first group—or in the sex distribution of cases. Although patients in the neoadjuvant treatment group had a lower Charlson comorbidity index, 3.8 (3.47–4.12) vs. 4.26 (4.03–4.48), it was not statistically significant. Regarding a more accurate detailing of the comorbidities, there were no differences in terms of cardiovascular, metabolic, renal or neurological diseases; there were only differences in terms of hypertension and patients in which surgery was performed having higher incidence or previous or synchronous tumors (19 patients (6.83%) versus 4 patients (3.8%)).

Regarding the type of neoadjuvant treatment in the first 5 years of the study, the preferred chemotherapy regimen was ECF and in the latter was FLOT. In 57 cases (54.28%), FLOT regimen was used; in 46 cases (43.8%), four cycles of chemotherapy were administered; and in 11 cases (10.47%), three cycles were administered based on the clinical evolution of the patients. ECF regimen was used in 40 cases (38.1%), three cycles in 28 cases (26.7%) and six cycles in 12 cases (5.7%). The remaining cases received DCX protocol. Based on the localization of the tumor, there was a tendency of initiating neoadjuvant chemotherapy for tumors located at the esogastric junction in 17 cases (16.2%) versus 23 cases (8.3%) or in the upper part of the stomach, which was respectively 18.1% versus 11.9%.

There were 190 total gastrectomies; they were more frequently performed in the neoadjuvant group (70 patients (66.7%) versus 122 patients (43.9%)), as shown in Table 2. In those cases, splenectomy was performed in 77 cases (40.5%); the indications for splenectomy were: oncological radicality, direct invasion or iatrogenic lesions. During the surgical exploration, 191 patients presented locally advanced tumors, a slightly higher percentage than in the neoadjuvant group (33.3% vs. 24.73%), and in 57 (14.9%) cases it required a multivisceral resection (besides splenectomy): pancreatectomy in 32 cases, liver resection in 15 cases or colon resection in 10 cases. There were 12 patients with synchronous tumors: in three cases a nephrectomy was performed for renal cancer, four colectomies for synchronous colon cancer, three small bowel resections for gastrointestinal stromal tumors and, in two cases, an adnexectomy for borderline ovarian tumor.

The perioperative evolution of the patients is summarized in Table 3. The overall incidence of severe postoperative complications (grade III or above in the Dindo Clavien scale) was 15.15% (58 cases). Although the anastomotic fistulas were more frequent in the surgery-first group with 23 cases (8.3%) compared to three patients (2.9%) in the neoadjuvant group, the result were not statistically significant. There was no difference in terms of duodenal stump fistula percentages, severe postoperative hemorrhage that required surgical intervention, abdominal collection or sepsis. Thirty-three patients developed medical complications, most commonly cardiovascular and pulmonary complications, with no differences between the two subgroups.

The management of anastomotic fistulas, both esojejunal and duodenal stump fistulas, consisted of conservatory treatment; surgical reintervention was only required in three cases of duodenal stump fistula and one case of esojejunal fistula due to the presence of local peritonitis. There were 13 cases in which surgical reintervention was required: in five cases for postoperative severe hemorrhage, bowel obstruction in three cases, drainage of peritoneal collection in three cases, and peritonitis in two cases. Postoperative 30-day mortality rate was 3.1% (12 cases) with equal statistical distribution in-between the two groups. The cause of death in patients with neoadjuvant treatment was sepsis due to esojejuanl fistula in one case and myocardial infarction in one case. For the surgery-first group the cause of death was abdominal sepsis due to esogastric fistula in three cases, pulmonary complications in two cases, myocardial infarction in two cases, ischemic stroke in two cases and lower limb ischemia in a patient with severe generalized atherosclerosis.

We also compared immediate postoperative complications as a short analysis regarding results between the first and latter 5 years: first 5 years—from a total of 181 cases, 37 underwent NAT, with three patients presenting with postoperative complications and 143 patients with up-front surgery, of whom 21 presented with postoperative complications (*p* = 0.43). In the last 5 years—from a total of 384 cases, 68 underwent NAT, with seven patients presenting with postoperative complications and 127 patients with up-front surgery, of whom 27 presented with postoperative complications (*p* = 0.11).

We performed a univariate analysis of factors involved in the occurrence of complications (Table 4). On multivariate logistic regression, only the PNI with an OR = 1.1 (0.89–1.31) and the presence of a neurological disease with an OR = 3.01 (1.12–8.05) were correlated with the occurrence of a postoperative complication.

Histopathological results are summarized in Table 5. A complete clinical response after neoadjuvant treatment was obtained in 10 cases; all of those patients presented tumors of the upper part of the stomach or of the gastroesophageal junction. There were no differences in the mean number of resected lymph nodes between the two subgroups; the highly statistically significant difference was the mean number of positive lymph nodes (5.74 vs. 7.99). Regarding the final pathological staging, there were no differences for stage I, IIb, IIIa and IIIc. There were 14 cases of patients in which pathological examination showed microscopic peritoneal carcinomatosis that was unrecognized during surgery. 

## 4. Discussion

Despite the fact that the current guidelines, such as the European Society of Medical Oncology, recommend perioperative chemotherapy in patients with resectable >stage IB gastric cancer [17], this practice has now gained enough clinical support due to the possible enhanced postoperative complications risk for patients requiring radical resection. Therefore, the aim of this study was to assess the occurrence of postoperative severe complications in gastric cancer patients that received neoadjuvant treatment protocol and to compare the pathological results.

Based on the results of this study, we used neoadjuvant treatment in 105 patients (27.41%), a lower percentage than other published data. At the beginning of the study period the neoadjuvant treatment was recommended to patients that were unfit for surgery (for example due to severe malnutrition) or patients that presented locally advanced tumors in which an R0 resection was considered to be improbable. Of those patients, after the completion of chemotherapy, a clinical and imaging evaluation showed an improved general status with a good clinical response to treatment and the patient became a candidate to surgery. Due to the good postoperative results in terms of morbidity, mortality and pathological response, we applied the same protocol to an increased percentage of patients; in the last two years approximatively 75% of patients received perioperative chemotherapy. The results of this study show that there were no differences regarding age, sex, or the presence of severe comorbidities. The only difference was in terms of the incidence of hypertension, explained by a meticulous investigation of cardiovascular status prior to and after chemotherapy. Another interesting aspect was the presence of synchronous or previous malignancies: patients without neoadjuvant treatment had a significantly higher percentage (6.83% vs. 3.8%),which can be explained by the effect of the previous tumor treatment or the patient’s preference. No differences in terms of hemoglobin levels, albumin as an indicator of nutritional status, or CEA and CA19-9 markers were found.

The results of this study also showed that we used perioperative treatment for tumors located in the upper part of the stomach or at the gastroesophageal junction. This can be explained by the fact that the distal tumors of the stomach tend to be more symptomatic (mostly gastric outlet obstruction), and this was the explanation for the highest percentage of total gastrectomies in this subgroup.

Regarding the postoperative complications, we observed no differences in terms of duodenal stump fistula, hemorrhagic complications, or abdominal collections; the only difference, although not statistically significant, was the occurrence of esojejunal fistula (2.9% vs. 8.3%, *p* = 0.06). No difference in terms of medical complications was found. The results of our study conclude with the results of a meta-analysis [18] that included 20 studies with 1420 patients with neoadjuvant treatment and 1942 with surgery first, which showed no difference in total complications and 30-day mortality. However, this meta-analysis showed less reoperation and less anastomotic leakage in the neoadjuvant group. A Finnish nationwide retrospective study that included 2708 patients showed a percentage of neoadjuvant treatment of 16.4% without increased postoperative complications, either medical or surgical (OR 1.12; 95% CI 0.81–1.56) [11]. A Turkish retrospective study on 186 patients [19], of which 51 received neoadjuvant treatment, showed an overall 51.6% complication rate and 5.4% leakage rate, without any differences in patients with neoadjuvant treatment. Furthermore, the study showed that age, sex, tumor location, T and N stage, and type of surgery (total vs. subtotal gastrectomy) did not have an impact on the occurrence of postoperative complications. There are several serum inflammatory biomarkers involved in the occurrence of postoperative complications, such as: presepsin, procalcitonin or fibrinogen, composite inflammatory and nutritional ratios (lymphocyte-to-C-reactive protein ratio, C-reactive protein-to-albumin ratio or fibrinogen-to-albumin ratio), and serum levels of Butyrylcholinesterase. According to the literature, low levels of this enzyme in the first and third post-surgery days are associated with an increased risk for the development of SSIs, but not sepsis, in patients undergoing colorectal surgery. This is currently considered a supportive biomarker rather than a standalone predictor. It may be most useful when combined with other clinical and laboratory parameters in a multimodal risk assessment model [20].

One of the local effects of chemotherapy in locally advanced gastric cancer is the fibrosis induced by tumor necrosis, and this is especially visible in patients with enlarged metastatic lymph nodes in the region of celiac or splenic artery. This is an indicator of a good response to chemotherapy and can pose difficulties in identifying the dissection plane, which thus can lead to an increased risk of complications. The activation of fibroblast plays a key role in this process, and it can be influenced by the usage of anti-fibrotic drugs and inhibitors of TGF-beta [21]. Another consequence of peritumoral fibrosis is the strong adhesions between the stomach and the surrounding organs, and this can impose a multivisceral resection, even though the final pathological examination shows only fibrosis or inflammatory adhesions. In our study there were five patients in the neoadjuvant group that required multivisceral resection and final pathological examination showed only fibrosis.

Extended lymphadenectomy in gastric cancer is one of the most important factors for accurate staging and prognosis. The presence of lymph node metastasis represents an important factor for the oncological prognostic of the patient. Current recommendations include the removal of at least 16 lymph nodes for accurate staging. In our series of patients, none of them had fewer than 16 lymph nodes—the mean number was 35.34 (33.1–37.6)—without difference in-between the two subgroups. One of the statistically significant differences was in the mean number of positive lymph nodes, with lower numbers for the neoadjuvant group as a direct effect of chemotherapy.

This study has several drawbacks: first, the retrospective nature of this study might impair the accurate acquisition of data regarding postoperative complications. The second drawback is the inclusion of a relatively small percentage of patients that received chemotherapy, including the fact that at the beginning of the study the patients received different chemotherapy regimens, and only in the final years was FLOT regimen the standard for perioperative treatment. Another limitation of this study is the fact that it was focused only on early postoperative outcomes, and it ignored the oncological outcomes such as survival, long-term complications and quality of life.

This study’s results conclude with the previously published retrospective studies [14,19,22] and have the advantage of the inclusion of a continuous series of patients, with a relatively similar diagnostic protocol. Surgical treatment involved a limited number of surgeons that respected the same surgical protocol in terms of organ resection and lymphadenectomy, and this is one of the strongest points of this study. We can affirm that all the patients received a standard resection and a standard D2 lymphadenectomy.

## 5. Conclusions

To our knowledge our study represents one of the largest studies from Eastern Europe regarding the early outcomes following perioperative treatment for gastric cancer in a single tertiary center. The results of our study support the safety of neoadjuvant treatment for resectable gastric cancer patients. We strongly support personalized treatment based on a multidisciplinary tumor board with dedicated surgeons and oncologists and the centralization of cases in tertiary centers where the patients could benefit from this approach. Furthermore, we plan to analyze long-term oncological results and to see the effect on overall survival, disease-free survival and quality of life.

## Figures and Tables

**Table 1:** Patients’ characteristics.

	Radical Gastrectomy N = 383 Patients (%)	Neoadjuvant Treatment Group N = 105 Patients (%)	Surgery First Group N = 278 Patients (%)	*p*-Value
Males	248 (64.75%)	67 (63.8%)	181 (65.1%)	
Females	135 (35.25%)	38 (36.2%)	97 (34.9%)	0.81
Age	66.8 (64.5–69.3)	64.99 (63.09–66.88)	67.83 (66.44–68.91)	0.44
CCI	4.1 (3.8–4.4)	3.8 (3.47–4.12)	4.26 (4.03–4.48)	0.131
Cardiac diseases	157 (40.9%)	43 (41%)	114 (41%)	0.91
Hypertension	146 (38.12%)	62 (59.04%)	84 (30.21%)	0.04
Diabetes mellitus	53 (13.8%)	16 (15.2)	37 (13.3%)	0.62
Pulmonary conditions	35 (9.1%)	11 (10.4%)	24 (8.6%)	0.55
Hepatic disease	54 (14.1%)	13 (12.4%)	41 (14.7%)	0.62
Chronic renal disease	60 (15.7%)	14 (13.3%)	46 (16.5%)	0.52
Neurological disease	27 (7.05%)	7 (6.7%)	20 (7.2%)	0.9
Previous/synchronous tumors	23 (6%)	4 (3.8%)	19 (6.83)	0.04
Hemoglobin	11.75 (10.92–12.58)	11.79 (10.87–12.72)	11.74 (11.39–12.09)	0.94
Albumin levels	4.24 (4.19–4.29)	4.28 (4.18–4.37)	4.21 (4.14–4.28)	0.3
CEA	6.8 (2.8–10.8)	5.85 (1.6–10.1)	7.29 (3.69–10.88)	0.65
CA19-9	37.95 (22.4–53.5)	38.39 (16.83–59.95)	37.53 (27.86–47.21)	0.93
PNI	50.65 (49.83–51.46)	50.9 (49.24–52.55)	50.55 (49.61–51.49)	0.7
PLR	199.11 (188.47–209.75)	162.57 (149.89–175.25)	212.91 (199.37–2226.46)	<0.001

CCI—Charlson comorbidity index, PLR—platelets-to-lymphocyte ratio, PNI—prognostic nutritional index.

**Table 2:** Tumor localization and operative characteristics.

	Radical Gastrectomy	Neoadjuvant Treatment	Upfront Surgery	*p* Value
Tumor localization				
Distal	172 (44.9%)	30 (28.6%)	142 (51.1%)	
Body	117 (30.6%)	33 (31.4%)	84 (30.2%)	
Upper stomach	52 (13.6%)	19 (18.1%)	33 (11.9%)	
Esogastric junction (Siewert III)	40 (10.4%)	17 (16.2%)	23 (8.3%)	0.04
Surgical approach				
Median laparotomy	379 (98.9%)	104 (99%)	275 (98.9%)	0.91
Thoracoabdominal approach	4 (1.1%)	1 (1%)	3 (1.1%)	
Peritoneal carcinomatosis	14 (3.7%)	4 (3.8%)	10 (3.6%)	
Local invasion	105 (27.4%)	35 (33.3%)	70 (2.5%)	0.4
Total gastrectomy	190 (49.6%)	70 (66.7%)	122 (43.9%)	
Subtotal gastrectomy	191 (49.9%)	35 (33.3%)	156 (56.1%)	<0.05
Splenectomy (only for total gastrectomy)	77 (40.5%)	24 (34.3%)	53 (43.4%)	0.47
Multiorgan resections	67 (17.5%)	20 (19%)	47 (16.9%)	0.65

**Table 3:** Postoperative complications.

	Radical Gastrectomy	NAT	Upfront Surgery	*p* Value
Esojejunal fistula	26 (6.8%)	3 (2.9%)	23 (8.3%)	0.06
Duodenal stump fistula	19 (4.9%)	4 (3.8%)	15 (5.4%)	0.6
Hemorrhage	7 (1.8%)	1 (0.9%)	6 (2.2%)	0.67
Collection	29 (7.6%)	4 (3.8%)	25 (8.9%)	0.12
Sepsis	31 (8.1%)	6 (5.7%)	25 (8.9%)	0.4
Medical complications	33 (8.6%)	6 (5.7%)	27 (9.5%)	0.3
Cardiac complications	32 (8.4%)	9 (8.6%)	23 (8.3%)	0.25
Pulmonary complications	30 (7.8%)	6 (5.7%)	24 (8.6%)	0.4
Hospital stay	13.7 (11.5–15.9)	13.9 (12.8–15)	13.1 (10.2–16)	0.78
Readmission rate	33 (8.6%)	10 (9.5%)	23 (8.27%)	0.69
Death	12 (3.1%)	2 (1.9%)	11 (3.9%)	0.49

**Table 4:** Univariate analysis of factors involved in the occurrence of complications.

	Radical Gastrectomy	NAT	Surgery First	*p* Value
Adenocarcinoma	371 (96.9%)	101 (96.2%)	270 (97.1%)	
NET-G3	12 (3.1%)	4 (3.8%)	8 (2.9%)	
G1	36 (9.4%)	5 (4.8%)	31 (11.2%)	0.01
G2	104 (27.1%)	22 (20.9%)	82 (29.5%)	0.4
G3	209 (54.6%)	52 (49.5%)	157 (56.5%)	0.3
Gx	26 (6.8%)	24 (22.9%)	2 (0.1%)	0.02
L1	284 (74.1%)	69 (65.7%)	215 (77.3%)	0.02
V1	216 (56.4%)	54 (51.4%)	162 (58.2%)	0.03
Pn1	187 (48.8%)	47 (44.8%)	140 (50.3%)	0.26
Resected lymph nodes	35.34 (33.1–37.6)	35.44 (32.51–38.37)	35.22 (33.43–37.01)	0.96
Positive lymph nodes	6.82 (4.80–8.84)	5.74 (4.15–7.32)	7.99 (6.64–9.34)	<0.001
Complete response pT0N0	-	10 (9.5%)	NA	
Stage I	36 (9.4%)	7 (6.7%)	29 (10.2%)	
Stage IIA	32 (8.4%)	8 (7.6%)	24 (8.63%)	
Stage IIB	61 (15.9%)	23 (21.9%)	38 (13.6%)	
Stage IIIA	43 (11.2%)	16 (15.2%)	27 (9.7%)	
Stage IIIB	111 (28.9%)	20 (19.1%)	91 (32.7%)	
Stage IIIC	76 (19.8%)	21 (20%)	55 (19.8%)	
Stage IV	14 (3.6%)	-	14 (5%)	

NA means data missing as it should since surgery first means no NAT and no possible complete response to NAT.

**Table 5:** Final pathological results.

	Complications n = 58	Without Complications n = 325	*p* Value
Age	69.23 (66.87–71.6)	66.52 (65.37–67.67)	0.06
Sex			
Males	47 (81.03)	202 (62.15%)	0.2
Females	11 (18.96%)	123 (37.84%)	
CCI	4.39 (3.9–4.88)	4.08 (3.88–4.29)	0.24
Cardiac disease	24 (41.37%)	133 (40.92)	0.93
Hypertension	14 (24.13%)	132 (40.61%)	0.31
Diabetes mellitus	11 (18.96%)	42 (12.92%)	0.22
Pulmonary conditions	5 (8.62%)	30 (9.23%)	0.82
Hepatic diseases	2 (%)	52 (16%)	0.02
Chronic renal diseases	6 (3.44%)	52 (16%)	0.22
Neurological diseases	8 (13.79%)	19 (5.84%)	0.05
Previous synchronous tumors	3 (5.17%)	20 (6.15%)	0.99
Neoadjuvant treatment	10 (17.24%)	48 (14.76%)	0.059
Hemoglobin	11.46 (10.74–12.18)	11.84 (11.05–12.62)	0.69
Albumin levels	4.12 (3.98–4.27)	4.28 (4.23–4.34)	0.02
PNI	47.14 (44.16–50.11)	51.28 (50.49–52.07)	0.0003
LPR	228.56 (193.1–264.01)	193.86 (183.03–204.68)	0.02
CEA	7.04 (2.2–11.89)	6.84 (3.49–10.2)	0.95
CA 19-9	55.09 (29.67–80.51)	33.5 (24.03–42.97)	0.06

## Data Availability

The data supporting the findings of this study are available from the corresponding author upon reasonable request, subject to applicable privacy regulations and legal restrictions.

## Abbreviations

The following abbreviations are used in this manuscript:

NAT	Neoadjuvant Treatment
US	Upfront Surgery
HER-2	Human Epidermal Growth Factor Receptor 2
PD-L1	Programmed Death-Ligand 1
MSI	Microsatellite instability
MMR	Mismatch Repair
FLOT	Fluorouracil, leucovorin, oxaliplatin and docetaxel
ECF	Epirubicin, Cisplatin, and Fluorouracil (5-FU)
5-FU	5-Fluorouracil
DCX	Docetaxel, cisplatin and capecitabine
PET	Positron Emission Tomography
TNM	Tumor, Node, Metastasis classification
pTNM	Pathological Tumor Stage
AJCC	American Joint Committee on Cancer
MDT	Multidisciplinary Team
CEA	Carcinoembryonic Antigen
CA19-9	Carbohydrate Antigen 19-9
NET-G3	Grade 3 Well-Differentiated Neuroendocrine Tumor
G1-3,X	Histological differentiation grade
V1	Histological vascular invasion
L1	Histological lymphatic invasion
Pn1	Histological lymphatic invasion
SD	Standard Deviation
CI	Confidence Interval
CCI	Charlson comorbidity index
PLR	Platelets to lymphocyte ratio
PNI	Prognostic nutritional index
